# Supplementary material for: Stepwise activation mechanism of the scramblase nhTMEM16 revealed by cryo-EM
Source: eLife. 2019 Feb 21;8:e44364. doi: 10.7554/eLife.44364 (PMC6414200; doi:10.7554/eLife.44364)
Supplement: Transparent reporting form [file elife-44364-transrepform.pdf]

eLIFE
1st Floor
24 Hills Road
Cambridge CB2 1JP, UK
P 01223 855340
W elifesciences.org
T @elife

eLife's transparent reporting form

We encourage authors to provide detailed information *within their submission* to facilitate the interpretation and replication of experiments. Authors can upload supporting documentation to indicate the use of appropriate reporting guidelines for health-related research (see EQUATOR Network), life science research (see the BioSharing Information Resource), or the ARRIVE guidelines for reporting work involving animal research. Where applicable, authors should refer to any relevant reporting standards documents in this form.

If you have any questions, please consult our Journal Policies and/or contact us: editorial@elifesciences.org.

Sample-size estimation

- You should state whether an appropriate sample size was computed when the study was being designed
- You should state the statistical method of sample size computation and any required assumptions
- If no explicit power analysis was used, you should describe how you decided what sample (replicate) size (number) to use

Please outline where this information can be found within the submission (e.g., sections or figure legends), or explain why this information doesn't apply to your submission:

- The sample size for cryo-EM analysis was chosen to obtain the best possible resolution for each dataset. The detailed procedure is outlined in materials and methods section on cryo-EM data collection and image processing
- No sample size was computed when the study was being designed
- No statistical method of sample size computation was used
- Scrambling experiments were generally repeated with samples obtained from independent purification and reconstitution experiments. Errors were calculated as s.e.m. and are shown in all figures containing scrambling experiments data.
- This information can be found in materials and methods (Reconstitution into the liposomes and scrambling assay).

Replicates

- You should report how often each experiment was performed
- You should include a definition of biological versus technical replication
- The data obtained should be provided and sufficient information should be provided to indicate the number of independent biological and/or technical replicates
- If you encountered any outliers, you should describe how these were handled
- Criteria for exclusion/inclusion of data should be clearly stated
- High-throughput sequence data should be uploaded before submission, with a private link for reviewers provided (these are available from both GEO and ArrayExpress)

Please outline where this information can be found within the submission (e.g., sections or figure legends), or explain why this information doesn't apply to your submission:

eLife Sciences Publications, Ltd is a limited liability non-profit non-stock corporation incorporated in the State of Delaware, USA, with company number 5030732, and is registered in the UK with company number FC030576 and branch number BR015634 at the address 1st Floor, 24 Hills Road, Cambridge CB2 1JP | August 2014

- Number of performed experiments is included in the figure legend.
- For lipid scrambling data, number of biological replicates refers to the number of independent protein reconstitutions. Each experiment was repeated on separate independent protein purification and reconstitution events.
- No obvious outliers regarding the particular behavior of independent biological replicates have been encountered and only outliers originating for technical reasons were excluded.
- No high-throughput sequencing data were involved in this manuscript
- Traces depicted in Figure. 7, Figure 1-figure supplement 1a,c, Figure 7-figure supplement 1, show averages of three technical replicates, errors are s.e.m. Traces depicted in Figure 1-figure supplement 1b show the average of two technical replicates, error shows the range.
- For lipid scrambling, all experiments in E. coli polar lipids/egg PC used for the characterization of wt and mutants were repeated in at least three independent purifications and reconstitutions with similar results.
- Reconstitutions in soybean polar lipids and nanodisc lipids depicted in Figure 1-figure supplement 1b,c were performed once.
- This information can be found in materials and methods section (Reconstitution into the liposomes and scrambling assay) and in figure legends for Figure 1 – figure supplement 1, Figure 7, Figure 7 – figure supplement 1

Statistical reporting

- Statistical analysis methods should be described and justified
- Raw data should be presented in figures whenever informative to do so (typically when N per group is less than 10)
- For each experiment, you should identify the statistical tests used, exact values of N, definitions of center, methods of multiple test correction, and dispersion and precision measures (e.g., mean, median, SD, SEM, confidence intervals; and, for the major substantive results, a measure of effect size (e.g., Pearson's r, Cohen's d)
- Report exact p-values wherever possible alongside the summary statistics and 95% confidence intervals. These should be reported for all key questions and not only when the p-value is less than 0.05.

Please outline where this information can be found within the submission (e.g., sections or figure legends), or explain why this information doesn't apply to your submission:

- For cryo-EM maps, the resolution was estimated with the gold-standard Fourier shell correlation between two independently refined half-maps. For details, see materials and methods.
- For the scrambling assay, this information can be found in materials and methods section (Reconstitution into the liposomes and scrambling assay) and in figure legends for Figure 1 – figure supplement 1, Figure 7, Figure 7 – figure supplement 1.

(For large datasets, or papers with a very large number of statistical tests, you may upload a single table file with tests, Ns, etc., with reference to sections in the manuscript.)

Group allocation

- Indicate how samples were allocated into experimental groups (in the case of clinical studies, please specify allocation to treatment method); if randomization was used, please also state if restricted randomization was applied
- Indicate if masking was used during group allocation, data collection and/or data analysis

Please outline where this information can be found within the submission (e.g., sections or figure legends), or explain why this information doesn't apply to your submission:

Individual reconstitution experiments were treated as separate groups, in which wt protein was compared to mutants. For details, see materials and methods line 424 – 427.

Additional data files ("source data")

- We encourage you to upload relevant additional data files, such as numerical data that are represented as a graph in a figure, or as a summary table
- Where provided, these should be in the most useful format, and they can be uploaded as "Source data" files linked to a main figure or table
- Include model definition files including the full list of parameters used
- Include code used for data analysis (e.g., R, MatLab)
- Avoid stating that data files are "available upon request"

Please indicate the figures or tables for which source data files have been provided:

N/A
